# Localization of Waves in Merged Lattices

**DOI:** 10.1038/srep31620

**Published:** 2016-08-18

**Authors:** G. Alagappan, C. E. Png

**Affiliations:** 1 Department of Electronics and Photonics, Institute of High Performance Computing, Agency for Science, Technology, and Research (A-STAR), 1 Fusionopolis Way, #16-16 Connexis, 138632 Singapore

## Abstract

This article describes a new two–dimensional physical topology–*merged lattice*, that allows dense number of wave localization states. Merged lattices are obtained as a result of merging two lattices of scatters of the same space group, but with slightly different spatial resonances. Such merging creates two–dimensional scattering “beats” which are perfectly periodic on the longer spatial scale. On the shorter spatial scale, the systematic breakage of the translational symmetry leads to strong wave scattering, and this causes the occurrences of wave localization states. Merged Lattices promises variety of localization states including tightly confined, and ring type annular modes. The longer scale perfect periodicity of the merged lattice, enables complete prediction and full control over the density of the localization states and its’ quality factors. In addition, the longer scale periodicity, also allows design of integrated slow wave components. Merged lattices, thus, can be engineered easily to create technologically beneficial applications.

Localization is a key concept in wave physics that enables control and manipulation of wave propagations. A periodic lattice of scatters with a complete bandgap for wave propagations in general is able to localize wave corresponding to bandgap frequencies if a systematic single or an extended disorder (defects) is introduced in the lattice^1^,^2^,^3^,^4^,^5^,^6^. There are also some special periodic geometries such as Lieb^7^,^8^,^9^,^10^,^11^,^12^ and Kagome^13^,^14^,^15^,^16^,^17^ lattices, which permit wave localizations without the presence of any defects. The unit cells of such lattices naturally allow destructive waves interference, enabling localization. Apart from the periodic topologies, quasi–periodic structures^18^,^19^,^20^,^21^,^22^ and random structures^23^,^24^,^25^,^26^,^27^,^28^,^29^,^30^,^31^,^32^,^33^ have been intensively studied for wave localizations. Random dielectric structures exhibiting strong wave diffusion is able to localize wave if the mean free path of the diffusion is equal or smaller than *λ*/2*π*, where *λ* is the wavelength. Such transformation from a diffusion state to a localized state is the direct consequence of the wave interference, and it is well known in the name of Anderson localization. Though, Philips Anderson first predicted such localization for electronic wavefunctions^23^, now Anderson localization is an ubiquitous phenomenon in wave physics, and it has been demonstrated for various wave topologies such as light in semiconductor dielectric powders^25^, light in photonic crystals with random disorders^24^,^26^, light in complex optical communication cluster^27^, light in disorder fiber beams^28^, microwaves in random copper tubes filled with metallic and dielectric spheres^29^, Bose Einstein condensates in random optical lattices^30^, acoustic waves in glasses^31^, acoustic waves in percolation systems^32^ water waves in random underwater structures^33^, etc.

Apparently, new topologies lead to new physics, and paves the way for a new technological exploration. In this article, using light wave as an illustration, we introduce a new paradigm of wave localization topology that possesses double spatial resonances. Doubly resonant systems such as mediums with electromagnetic induced transparencies^34^,^35^,^36^, and their optical^37^,^38^,^39^,^40^, and plasmonic^41^,^42^ analogues are typically made of two physical sub-systems (usually resonators or atomic states) of slightly different resonant frequencies. These systems are known for their coherent interference effects, especially they can induce transmission in the originally opaque or reflective state. We borrow this idea to create majestic, large number of light localization states in two dimensional (2D) periodic lattices that has slightly different Bragg resonances.

Two lattices of slightly different periodicities but with the same space group can be merged together to produce a new lattice of the same space group but periodic on the longer spatial scale. For an example, consider two square lattices of periods *a* and *ra* (*r* > 1, and is a rational number close to 1). These two square lattices can be merged together to generate a merged lattice (ML) that is also a square lattice, but periodic on the longer spatial scale with a period *Ra*. Here, *R* is the least integer multiple of *r*. The square lattice dielectric functions with periods *a* and *ra*, have fundamental spatial resonances when the absolute value of the reciprocal lattice vectors equal 2*π*/*a* and 2*π*/*ra*, respectively. The closer these spatial resonances are, the longer the resulting 2D spatial dielectric beat. The symmetrically allowed closest proximity between the spatial resonances is 2*π*/*Ra* [i.e., absolute value of one reciprocal lattice vector of the ML]. This requirement constraints *r* as *r* = *R*/(*R*−1) {or equivalently *R* = *r*/(*r*−1)}.

Figure 1(a) to 1(h) illustrates the basic principles of creating MLs. Figure 1(c,g) show the primitive unit cells (i.e., the 2D dielectric beats) of MLs with *R* = 3 and 5, respectively. These primitive unit cell of the ML is obtained by combining *R* primitive unit cells of the lattice with period *a* {Fig. 1(a) [R = 3] and 1(e) [R = 5]}, with *R*−1 primitive unit cells of the lattice with period *ra* {Fig. 1(b) [R = 3] and 1(f) [R = 5]}. Note that (*R*−1) × *ra* = *Ra*, and when merging two lattices, the motifs at each lattice site is also merged [see Fig. 1(g,h)]. The primitive unit cells of MLs shown in Fig. 1(c,g) are not unique. Another set of primitive unit cells are shown in Fig. 1(d,h) for *R* = 3 and 5, respectively. The positions of these two different primitive unit cells [Fig. 1(c,d)] are illustrated in the Fig. 2 for *R* = 3.

The single 2D dielectric beat [i.e., the primitive unit cell of the ML] itself has no translational symmetries within, and hence it enables creation of large number of scattering loop that facilitates polychromatic, light localization. Unlike quasi–periodic dielectric structures^18^,^19^, or random dielectric structures^25^,^26^,^27^,^28^, such light localizations in dielectric MLs are completely predictable (and therefore controllable) using photonic band structures. The density of localization states and their quality factors scale as a function of *R*. Merged lattices (MLs) lie in between the two extremes of completely random system (where Anderson localization^23^,^24^,^25^,^26^,^27^,^28^ prevails), and the conventional periodic system of photonic crystals^4^,^5^. In the MLs, the periodicity prevails on the longer spatial scale (spatial distance >*Ra*). The translational symmetry is completely broken on the spatial scale <*Ra*. Localization in ML takes place within this shorter spatial scale, for wavelengths on the order of fractions of *a*.

MLs for light wave will be useful to create multimode miniature lasers^43^, efficient quantum computing^44^, efficient solar energy harvesting^45^, and enhanced non-linear optical interactions^46^. In addition to that, as MLs are perfectly periodic with the period *Ra*, thus they can be engineered easily to create defect paths enabling integrated slow light components, and photonic chips.

## Results

Before we discuss the optical properties of the 2D structures created from MLs, let us briefly review the band structure of the conventional 2D square lattice photonic crystal (PC) with the period *a*. For the sake of numerical illustration, assume the PC is made of circular silicon rods (refractive index, *n* = 3.4, radii of the rods equal to 0.15*a*) in an air ambience. Figure 3(a) shows the schematic of the non-primitive unit cell of the 2D PC with the length *Ra*. Rod PC with a square lattice is favorable for the transverse magnetic (TM) polarization [electric field along the axis of the rod]^5^. The photonic band structure of the 2D PC for light wave with the TM polarization, can be obtained by solving Maxwell’s equations using a plane wave expansion methodology^47^,^48^,^49^ (see the methods sections, for the details of the calculation). Figure 3(c) shows the result of calculation for a primitive unit cell (*R* = 1), and as we can see clearly see, the PC exhibits a large bandgap between the bands 1 and 2 for the TM polarized light. Figure 3(d–f) display photonic band structures for the non-primitive unit cells. The vertical axes in the figures represent normalized frequencies (*a*/*λ*). As all unit cells belong to the same 2D PC, the corresponding bandgaps remains exactly the same. The unit cell length of the non-primitive unit cell is *Ra*, and its’ Brillouin zone (BZ), as shown in Fig. 3(b), is shrunk *R* times in each direction. Therefore, the corresponding photonic band structures of the non-primitive unit cells [Fig. 3(d–f)] can be obtained by folding the original bands of the primitive cell [Fig. 3(c)], *R*^2^ times into the BZs of the non-primitive unit cells^6^,^49^. In other words, each band in the band structure of the primitive unit cell, becomes *R*^2^ folded bands in the band structure of the non-primitive unit cell. For a conventional PC, it is well known that the group velocities for the band edge modes are small, and the bands flatten towards the photonic bandgap frequencies. Therefore, the folded bands of the conventional PC in the proximity of photonic bandgaps are flatter [for example, see the folded band diagrams of Fig. 3(d–f)]. The mode pattern corresponding to the band edge mode is basically a standing wave that extends through the entire 2D lattice^5^. Thus, the band edge modes of the conventional PC are not spatially localized.

Note that the folded band structure of the conventional PC for *R* > 1 [Fig. 3(d–f)], possess many degenerate point and degenerate bands. Such degeneracies accounts for the finite translational symmetries within the corresponding non-primitive unit cells. These degeneracies will be lifted up, when the translational symmetries within the unit cell becomes broken. As we shall see, this is the exactly the case for the MLs, which breaks the translational symmetries of the original lattice before the merging.

**Figure 1:**
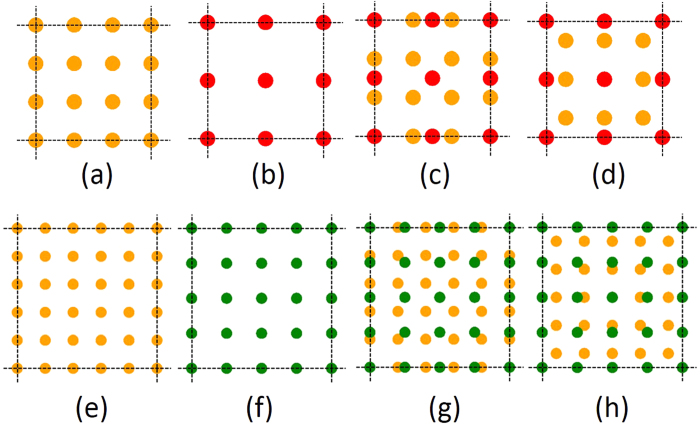
Merging of two square lattices of periods *a* and *ra*. Here, *r* = *R*/(*R*-1), with *R* is a integer greater than 2. (**a–d**) Merging for *R* = 3, (**e–h**) Merging for *R* = 5. (**a,e**) *R* periods of square lattice with period *a*. (**b,f**) (*R*-1) periods of square lattice with period *ra*. (**c**) One primitive unit cell of the lattice obtained from merging lattice in (**a**) with lattice in (**b**). (**g**) One primitive unit cell of the lattice obtained from merging lattice in (**e**) with lattice in (**f**). (**d,h**) Alternative primitive unit cells of the merged lattices.

**Figure 3:**
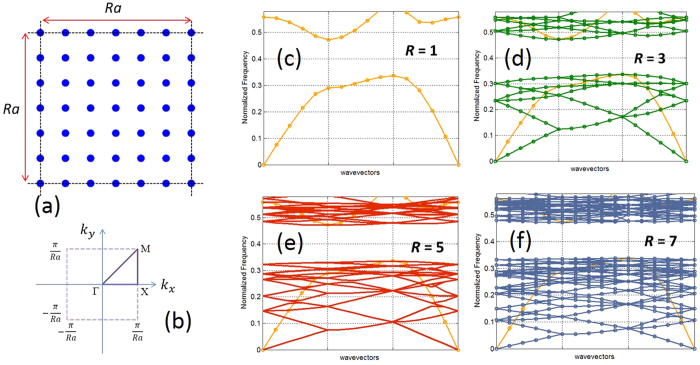
(**a**) Non-primitive unit cell of the square lattice with a length *Ra*. (**b**) BZ (dashed line) of the square lattice. The dark solid line represents the irreducible part of the BZ. Photonic band structures [TM polarization] for the 2D square lattice PC made of silicon rods in air matrix, for various unit cell lengths (**c**) *R* = 1 [orange] (**d**) *R* = 3 [green] (**e**) *R* = 5 [red] (**f**) *R* = 7[blue]. In (**d**) to (**f**), the band structures in the orange color is for *R* = 1 [reproduced from (**c**) for a comparison].

**Figure 4:**
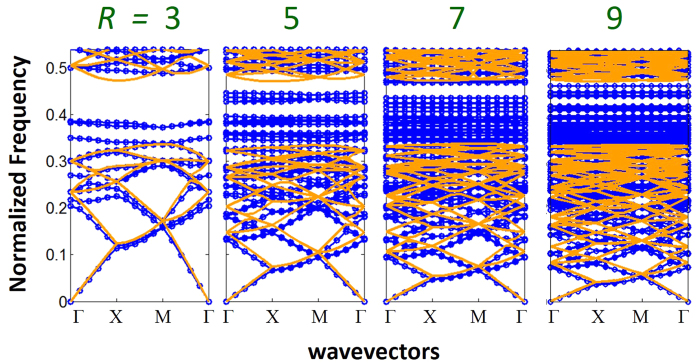
Photonic band structures of the merged lattices [blue] for *R* = 3, 5, 7 and 9. The band structures in the orange color are the folded band structures of the conventional square lattice PC with a non-primitive unit cell of length *Ra* [see Fig. 3(c–f)].

Now let us analyze the photonic band structures of MLs. Let assume MLs are created by merging the dielectric functions of two square lattice PCs [silicon rods in air ambience] with periods *a* and *ra* as described in Fig. 1. For the purpose of comparison with the band structures in Fig. 3, the radii of the rods are taken as 0.15*a* in both PCs. Figure 4 exhibits photonic band structures of the MLs for *R* = 3, 5, 7 and 9, respectively [see the methods sections, for the details of the calculation]. In the same diagram, we have also plotted the folded band structure of the conventional PC [i.e., lattice with period *a*; the corresponding folded band structures are also shown in Fig. 3(d–f)] with non-primitive unit cells of the length *Ra*. As we can from these figures, in the long wavelength limit, the bands of the ML look similar to the folded bands of the conventional PC. For this spectral region, the wavelengths are much larger than *a*, and as both ML and the conventional PC exhibit similar long range translational symmetries, it is not surprising to find their bands are similar. On the other hand, for the spectral region closer to the bandgap of the conventional PC, for which the wavelengths are on the order of fractions of *a*, the original translational symmetries in the non-primitive unit cells are completely broken. This induces coupling between the various folded bands of the conventional PC [Fig. 3(d–f)]. The coupling splits and flattens the folded bands, lifts–up the degeneracies, and pushes them into the bandgap region [Fig. 4]. From Fig. 4, we can clearly see that, MLs have dense number of flat bands in their band structure right at the vicinity of the bandgap region of the conventional PC. These flat bands occur for wavelengths (*λ*) on the order of fractions of *a* [see Fig. 4 for the normalized frequencies (*a*/*λ*) of the flat bands]. The number of flat bands in ML increases as *R* increases, and the bandwidth (frequency span of the band) of each flat band decreases as *R* increases.

**Figure 5:**
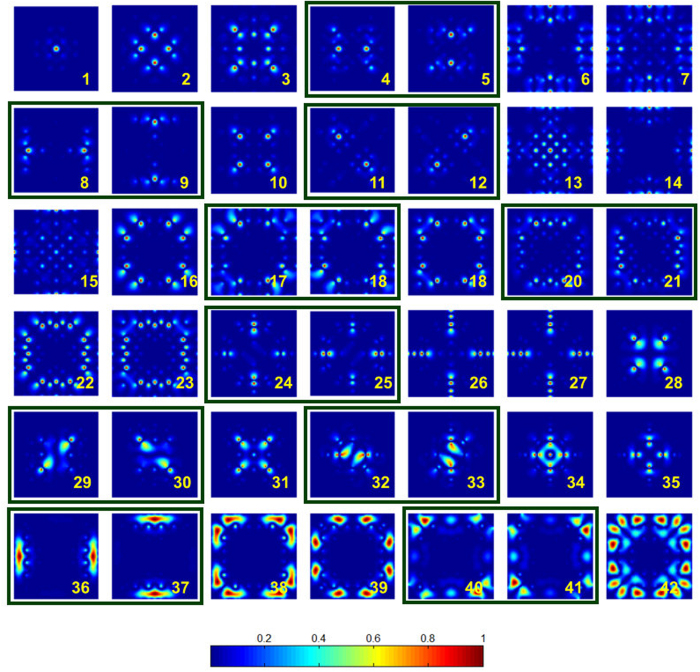
Mode field density (|**E**|^2^) at the Γ point of the merged square lattice with *R* = 7. The mode patterns correspond to the flat bands *R*^2^ + 1 to *R*^2^ + 42, and plotted for one primitive unit cell. The boxed pair of modes corresponds to the pair of bands with the degenerate frequencies.

Flat band has a vanishing group velocity, and it is the key signature of a slow mode^50^,^51^,^52^,^53^,^54^. Thus, the dense flat bands shown in Fig. 4 guarantees a varieties of localized modes in MLs. Figure 5 illustrates the localized mode patterns [mode filed density] for *R* = 7 at the Γ point of the bands (*R*^2^ + 1) to (*R*^2^ + 42). As we can see from this figure, ML creates variety of configurations that enables localization of light. Quite clearly, the breaking of translational symmetry in the ML (on the spatial scale <*Ra*), allows creation of multiple scattering loops within its primitive unit cell, and therefore realizes a large number of wave localization states. The density of the localized modes in ML scales as a function of *R*. As *R* increases, the spatial region of the broken translational symmetry, enlarges. This creates more opportunities for light localization.

Using group theory, the eigenmode at every band, for any given every **k** vector, can be uniquely identified with an irreducible representation (IR) of the symmetry group of the **k** vector^49^,^55^,^56^,^57^,^58^. In the reciprocal space, the high symmetrical **k** vectors of the square lattice are 
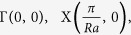
 and 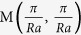 [see Fig. 3(b)]. In Schoenflies notation^55^,^56^, the symmetry group for these **k** vectors are respectively, *C*_4*v*_, *C*_2*v*_, and *C*_4*v*_. The symmetry representation for the group *C*_4*v*_ consists four non-degenerate IRs, and one doubly degenerate IR. On the other hand, the symmetry representation for the group *C*_2*v*_ consists four non-degenerate IRs. As any band is a continuous path (or a surface) in the reciprocal space, the adjacent bands are bound to touch each other if the bands possesses any degenerate IR at the symmetrical **k** vectors. In the square lattice, such touching will only occur at Γ and Μ points, because only these **k** vectors have doubly degenerate IRs. The degenerate modes at the Γ point of the discussed ML are boxed together in Fig. 5.

**Figure 6:**
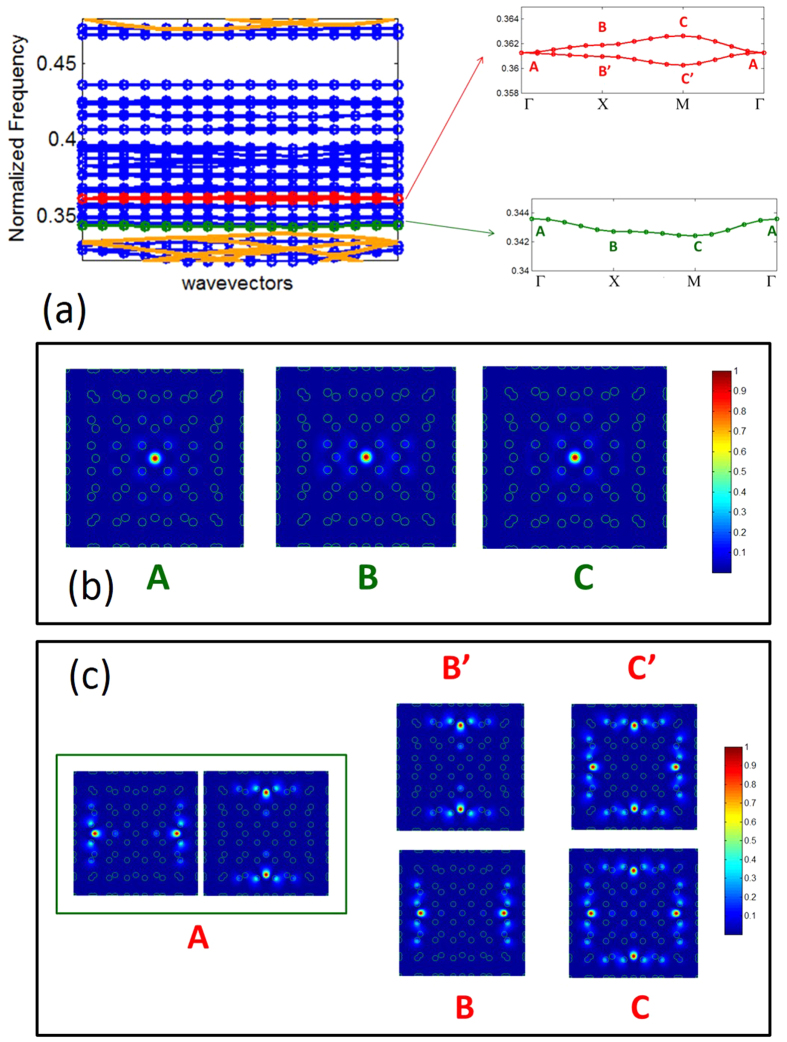
(**a**) Left: Enlarged version of the band structure shown in Fig. 4 for *R* = 7. Right: Two different genre of flat bands. The first genre is flat band with no degenerate points [for example, 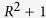 –th band (shown in green)], and the second is flat band with at least one degenerate point [for example, the *R*^2^ + 8 –th and *R*^2^ + 9 –th band (shown in red) touches each other at the Γ point]. (**b**) Mode pattern along the first genre of flat bands [*R*^2^ + 1 –th band is shown for an illustration]. (**c**) Mode pattern along the second genre of flat bands [*R*^2^ + 8 –th and *R*^2^ + 9 –th bands are shown for an illustration].

The knowledge on the degeneracy, allows us to categorize the flat bands into two important categories. The first category is flat bands with no degenerate points, and the second category is flat bands with at least one degenerate point. These two genre of flat bands have distinct mode dispersion. In general, the first category of flat bands displays more symmetrical mode than the flat bands from the second category. In Fig. 6, we illustrate examples of mode profiles for the two genre of flat bands.

**Figure 7:**
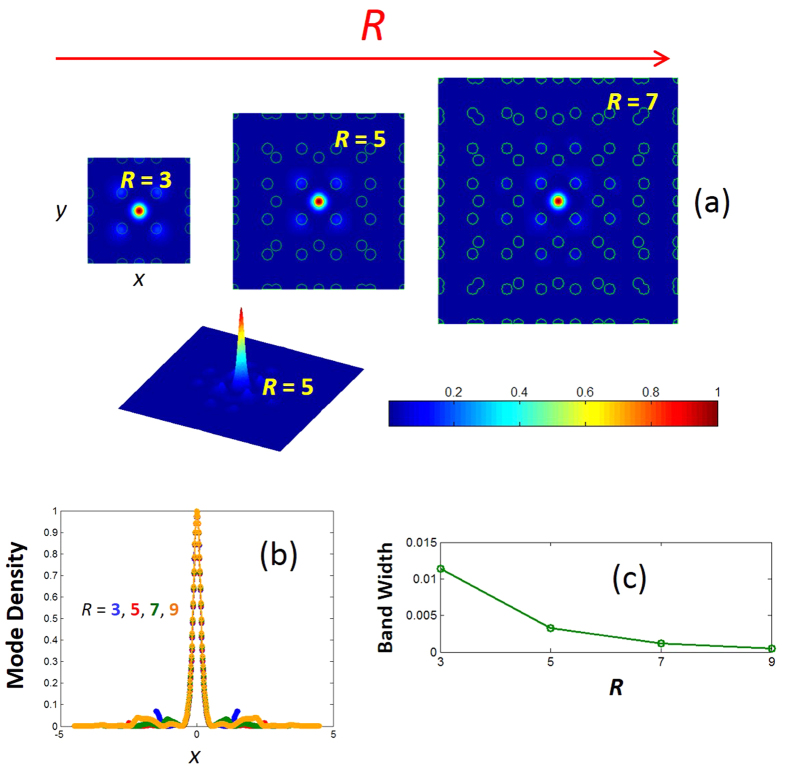
(**a**) Mode field density of the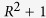 –th band (Γ point). (**b**) The horizontal {*x*} cross section of the mode field density for various *R*. (**c**) The frequency span (band width) of the *R*^2^ + 1 –th band as a function of *R*.

For an example of flat band with no degenerate points [i.e., the first type], let us take the *R*^2^ + 1 –th band. The enlarged plot of the frequency dispersion for *R* = 7 is shown in Fig. 6(a) [green color], and the corresponding mode profiles at the symmetrical **k** vectors are shown in Fig. 6(b). As can be seen from Fig. 6(b), the first genre of flat bands exhibits a nearly same mode profile (i.e., a small dispersion) along the **k** vector path. The electric field of the *R*^2^ + 1 –th band is sharply confined in the center of the unit cell (almost entirely in one rod). Figure 7(a) shows the evolution of the mode profile [*R*^2^ + 1 –th band, Γ point] as a function of *R*. In Fig. 7(b), we show the horizontal (*x*) cross section of the mode field density for various *R*. Figure 7(b) shows that the mode radius remains the same for all *R*. The calculated mode area is 0.34 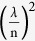. As we have said earlier, the flatness of the band increases as *R* increases, and this is quantified in Fig. 7(c) for the *R*^2^ + 1 –th band. As we can clearly see from the figure, the bandwidth (frequency span of the band) decreases almost one order, as *R* reduces from 3 to 9. As *R* increases, the length of the primitive unit cell increases, and therefore the evanescent coupling of localized to the adjacent unit cells decreases. This in turn results in a flatter dispersion^50^. A flatter band exhibits mode with higher quality factor. Given a fixed mode volume (area in 2D), and the higher quality factor as *R* increases, enhances Purcell factor and creates a favorable condition for variety of application in quantum computing and cavity quantum electrodynamics^44^,^59^.

Let us consider the *R*^2^ + 8 and *R*^2^ + 9 –th bands [Fig. 6(a) {red color}]. These pair of bands are degenerate at the Γ point, and hence belongs to the second genre of flat bands. The mode profiles of these bands at the Γ point consists of two independent eigenmodes as shown in the box A of Fig. 6(c). These degenerate mode splits along the path, Γ–X. The modes profiles at the X point are indicated as B’ and B for bands 8 and 9, respectively [see Fig. 6(a,c)]. As stated earlier, Γ point has *C*_4*v*_ point group symmetry, and therefore the mode must be invariant with respect to all symmetry operations of the group *C*_4*v*_. However, the individual modes in box A, on its own, does not have the *C*_4*v*_ symmetry [for example, the π/2 rotational symmetry is missing]. Thus, by symmetry requirement the two modes in box A must co-exist (i.e., degenerate). These individual mode possess a *C*_2*v*_ symmetry, and when they split along the path Γ–X, this symmetry is retained. This splitting is perfectly consistent with the symmetry requirement at point X, which has the *C*_2*v*_ symmetry.

**Figure 8:**
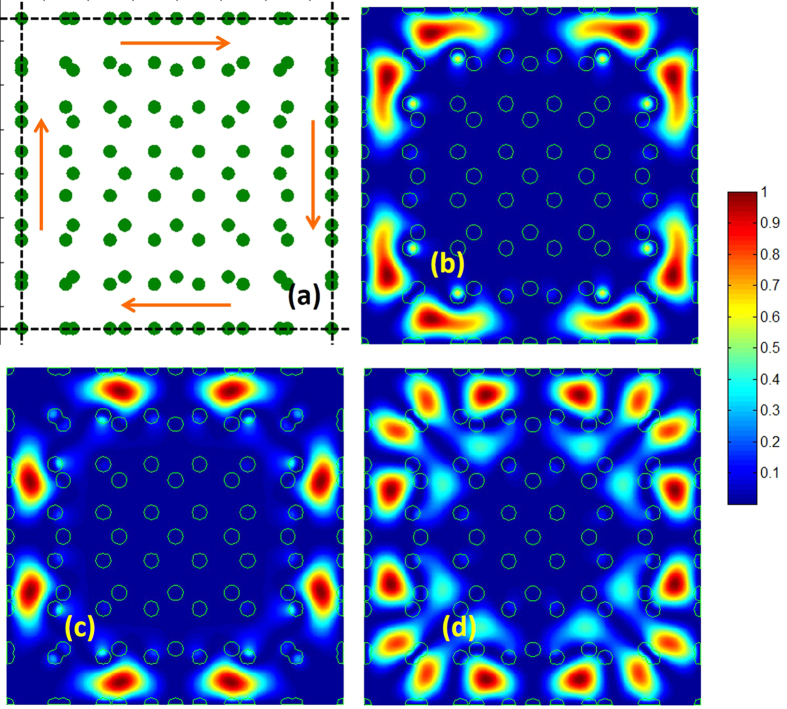
(**a**) The primitive unit cell for *R* = 7. The orange arrow shows the closed path for light localization. (**c–d**) The ring type of modes in the closed path of (**a**), correspond to the bands (**b**) 38 (**c**) 39, and (**d**) 42.

Figure 8 highlights a special group of modes in the ML that have ring shapes. These modes are localized in the closed path shown in Fig. 8(a). As oppose to the usual ring modes which are trapped in the dielectric region, these ring modes [Fig. 8(b–d)] in the ML are trapped in the air region. Therefore, these ring modes are robust with respect to material dispersion, and hence suitable for variety of applications in non-linear optics. Furthermore, the periodic nature [on the longer spatial scale] of the ML, creates a two dimensional array of such ring modes, similar to a two dimensional array of ring resonators^60^.

Wave localization states in a ML are different from the band edge modes of a conventional PC [although both have vanishing group velocities]. Unlike the band edge modes of the conventional PC (for which the standing wave modes extend through the entire lattice), the modes of MLs are spatially localized with smaller modal volumes (areas in 2D). For the conventional PC, the band edge mode is a result of coherent interference effects arising from the periodic arrangement of the scatters (i.e., Bragg scattering), and hence, periodicity is an essential prerequisite in observing the band edge modes. However, in the ML, flat band occur as a consequence wave localization due to the breaking in the translational symmetry, resulting from the merging. The periodicity *Ra*, is thus, not required to observe the localized modes of the ML (as they are not resulting from Bragg scattering).

The modes of ML are well localized within the primitive unit cell, and only the evanescent tail couples to the adjacent unit cell. Therefore, a single unit cell is sufficient to spatially confine light in ML. In fact, the mode patterns in Fig. 5 can be reproduced using a single primitive unit cell of the ML, assuming a non-periodic boundary condition such as perfectly matching layer boundary condition^61^.

The primitive unit cell of the ML also can be visualized as an optical resonator that supports large number of localized modes with finite and large lifetimes. The photonic band structure [Figs 4 and 6] is then, can be regarded as a display of the dispersion of these modes, when identical resonators arranged in a periodic fashion (i.e., the dispersion of the slow mode of the coupled optical resonators^50^). The photonic band structure also shows, how the degenerate mode of the optical resonator splits when identical resonators arranged in periodic fashion [for instance, see Fig. 6(c)].

## Methods

The photonic band structures [Figs 3, 4 and 6(a)], and the mode patterns [Figs 5, 6(b,c), 7(a) and 8(b–d)] are calculated using a plane wave expansion methodology. This method assumes a periodic boundary condition for the unit cell of the dielectric function, and Bloch theorem for the electric field. In the method, both electric field and the periodic dielectric functions are expanded in terms of plane waves, and substituted in the Maxwell’s equations. The resulting equations are combined as a matrix eigenvalue problem. In this paper, we used the freely available plane wave expansion solver, MPB^48^. The photonic band structure of the conventional lattice is obtained using 1024 plane waves. On the other hand, the photonic band structures, and the mode patterns of the MLs are obtained by employing (*n*_*resl*_*R*)^2^ plane waves, where *n*_*resl*_ = 64 is the resolution parameter used in MPB.

## Conclusion

In this article, we have presented the principles of two dimensional merged lattice constructions. Using light wave in square lattice as an example, we have demonstrated the salient wave localization properties of the merged lattice. We show, how the photonic band structure of the merged lattices evolves from the corresponding folded band structure of the conventional photonic crystals. The destruction of the translational symmetry induces strong coupling between the folded bands, and results in dense number of flat bands with variety of localized mode profiles. The mode profiles include sharp localized modes with small mode volumes, and ring modes trapped in the annular air region.

Although, the periodicity of merged lattice on the longer spatial scale is not required to see the localized modes of the merged lattice, the natural periodicity that it has, is certainly an added advantage. The periodic configuration of merged lattice in the longer spatial scale allows coupling of localized modes to the similar modes in the adjacent unit cells, forming a 2D coupled resonator system. In optics, such 2D coupled resonators are useful when constructing integrated slow light devices and circuits.

## Additional Information

**How to cite this article**: Alagappan, G. and Png, C. E. Localization of Waves in Merged Lattices. *Sci. Rep.* **6**, 31620; doi: 10.1038/srep31620 (2016).

## Figures and Tables

**Figure 2:**
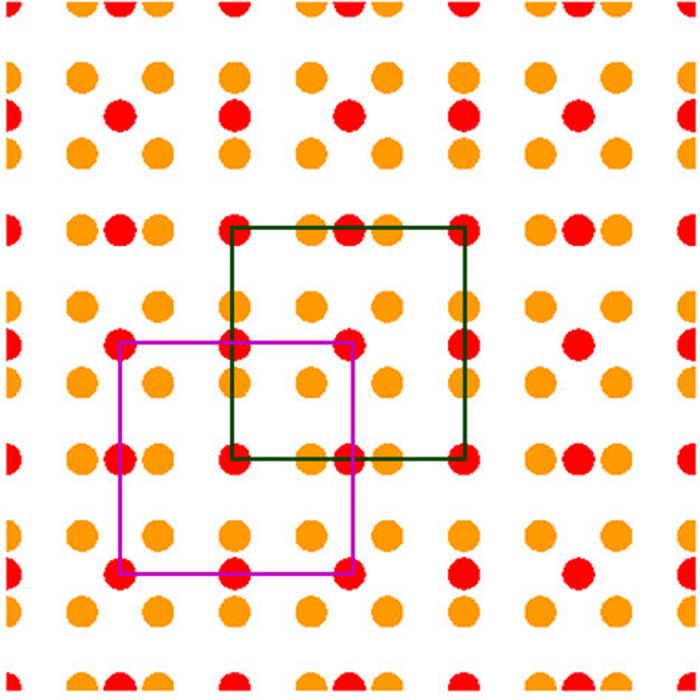
The relative positions of the two unique primitive unit cells for the case of *R* = 3 [primitive unit cell of Fig. 1(c) is shown in dark green color, and primitive unit cell of Fig. 1(d) is shown in purple color].
